# Modulatory Effect of Protein and Carotene Dietary Levels on Pig gut Microbiota

**DOI:** 10.1038/s41598-019-51136-6

**Published:** 2019-10-10

**Authors:** Rayner González-Prendes, Ramona Natacha Pena, Emma Solé, Ahmad Reza Seradj, Joan Estany, Yuliaxis Ramayo-Caldas

**Affiliations:** 1 Departament de Ciència Animal, Universitat de Lleida-Agrotecnio Centre, Lleida, 25198 Catalonia Spain; 2 Animal Breeding and Genomics Group, Wageningen University & Research, Droevendaalsesteeg 1, 6708 PB Wageningen, The Netherlands; 3 Animal Breeding and Genetics Program, IRTA, Torre Marimon, Caldes de Montbui, Catalonia Spain

**Keywords:** Applied microbiology, Microbiome, Microbial ecology

## Abstract

In this study we investigated the impact of dietary protein and carotene levels on microbial functions and composition during the last month of purebred fattening Duroc pigs. Fecal microbiota was characterized using 16S ribosomal RNA sequencing at two points of live, 165 (T1) and 195 (T2) days. From 70 to 165 days of age, 32 pigs were divided into two groups fed either a standard-protein (SP) or a low-protein (LP) diet. In the last month (165–195 days), all pigs received a LP diet, either carotene-enriched (CE) or not (NC). Significant differences were observed between T1 and T2 at Amplicon Sequences Variants (ASVs), phylum and genus levels. In T1 group, *Prevotella*, *Faecalibacterium* and *Treponema* were the genera most influenced by dietary protein, together with predicted functions related with the degradation of protein. In contrast, the CE diet did not impact the microbiome diversity, although 160 ASVs were differentially abundant between CE and NC groups at T2. Weak stability of enterotype clusters across time-points was observed as consequence of medium-term dietary interventions. Our results suggest that during the last month of fattening, dietary protein have a stronger effect than carotenes on the modulation of the compositional and functional structure of the pig microbiota.

## Introduction

The microbiota is a heterogeneous ecosystem composed of thousands of microbial species that influence on host physiological functions such as food intake, production of vitamins, metabolism, immune system activation, and resistance to infection^1^. Previous studies in pigs described the composition of microbiota at growing-finishing and fattening phases^2–6^. Modulations of the gut microbial community can have beneficial effects on pig’s health through direct stimulation of the immune system and also through microbiota-generated nutrients such as aminoacids, vitamins or short-chain fatty acids^7^.

As in humans, pig microbiota diversity starts being established at birth^2^ and reaches some stability at adulthood^2,5^. It has been postulated that microbiota could be modulated to improve the health or/and the productivity of pigs^7,8^. According to bacterial relative abundance, the pig gut microbial composition is structured in two well-defined enterotypes^2,9,10^ that remain more stable in post-weaning pigs than in younger animals^2^. This classification can change with the influence of many factors including the age of the animal, hygienic and health conditions and the diet^7,8^. For instance, in model animals, the level of protein and Vitamin A in feed can affect the gut bacterial ecosystem, which in turn can disturb the physiology of the host^11–14^. The amount of protein in the diet has a direct effect on gut microbiota with a positive correlation with the microbial diversity^15,16^. This correlation depends directly on the quantity and quality of the protein^15^. Protein-enriched diets results in longer transit time and a higher microbiota concentration in the large intestine as this section plays a relevant role in protein degradation into peptides and amino acids via extracellular bacterial proteases and peptidases^17^. In addition, low‐protein diets can be used to reduce the cost and environmental burdens of livestock production without affecting performance^18^. For instance, low protein diets can impact pig microbiota by decreasing microbial metabolites such as ammonia^19^. On the other hand, restriction on dietary vitamin A has been proposed as a dietary intervention to improve intramuscular fat content, a trait related to meat quality, in beef^20^ and pork. Provitamins A such as β-carotenoids are transformed into the active vitamin A mainly in the gut epithelial cells, a process that is enhanced by the action of colon microbiota over fat solubilisation^11^. Modified crops such as high-carotenoid corn can impact directly in the levels of vitamins A in the feed^21^. In a previous work we have shown the combinatory effect of dietary protein and vitamin A on intramuscular fat content and composition^22^. In the present study we question whether pig microbiota diversity has been affected by these dietary interventions and can relate to some of the phenotypic changes. Therefore, in this study we used 16S rRNA gene sequencing to evaluate the impact of different levels of protein and high-carotene diets on the fecal microbiota of 32 Duroc pig in terms of taxonomic and functional composition across two age-points during the fattening period.

## Results and Discussion

In this study 16S rRNA gene sequences from 64 fecal samples of Duroc pig collected in two time-points (T1, 165d of live and T2, 195d of live) were used to determine the influence of dietary protein (T1: SP, standard-protein vs LP, low-protein diets) and dietary carotenes (T2: CE, carotene-enriched vs NC, not carotene diet) on the microbiota composition. After quality control, a total of 3,286,123 contiguous sequences were retained to detect 3,141 Amplicon Sequences Variants (ASVs). In agreement with previous findings in Duroc pigs^4^, as well as in other pig breeds^2,9,10,23–25^, the most abundant phyla in both time-points were Firmicutes (55.16%, in T1 and 46.48%, in T2) and Bacteroidetes (33.58%, in T1 and 36.47%, in T2). In both time-points, the less abundant phyla were Proteobacterius (5–6%), Spirochaetes (2.3–6%) and Verrucomicrobia (0.9–1.3%). At genus level, the predominant genera were *Prevotella* (18.68%, in T1 and 14.96%, in T2) followed by the uncategorized group (5.23%, in T1 and 5.82%, in T2) and *Oscillospira* (4.56%, in T1 and 4.54%, in T2). The abundance of *Treponema* genus was higher (p = 1.16E05) in T2 (5.48%) than in T1 (2.40%).

### Dietary intervention affects fecal microbiota composition rather than diversity

Before estimating diversity indexes (alpha, beta and richness), samples were rarefied at 6,000 reads of depth to allow an equal depth in all samples. Observed similarities at taxonomic levels between time points were also reflected at diversity index level. There were no significant differences (p > 0.05) between the T1 and T2 groups, neither within each treatment group in T1 (SP vs LP) or T2 (CE vs NC). It is well known that diet, environment, lifestyle and resource availability can influence the composition and function of the gut microbiota^26–31^. However, similarly to our results, other groups have reported no changes in the ileal, cecal and colonic microbiota diversity between pigs fed with different inclusion level of protein^32,33^, which may be partially explained by the smaller difference (3%) of protein inclusion between diets in our and some of those studies^32,33^. In line with these results, reduction level of protein inclusion in piglets can result in lower intestinal ammonia concentration without significance changes of the bacterial community^34,35^. Indeed, in pigs, changes in dietary protein inclusion levels can affect microbiota composition rather than diversity, even during drastic dietary challenges such as those taking place at weaning^36^.

**Figure 1:**
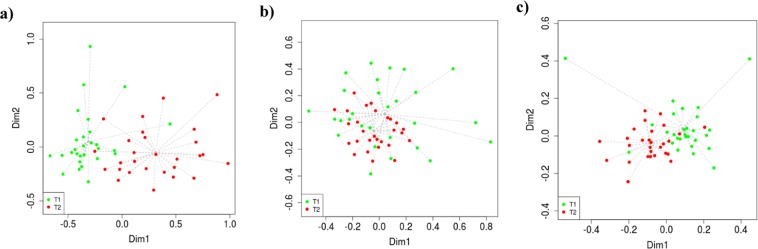
Non-Metric Multidimensional Scaling (NMDS) plots of bacterial communities at (**a**) Amplicon Sequences Variants, (**b**) phylum and (**c**) genus levels by samples taken at 165 days (T1) and 195 days (T2).

Despite the absence of relevant changes in the diversity indexes, compositional differences between groups were observed. According to the Non-metric multidimensional scaling (NMDS) and Multivariate analysis of variance (PERMANOVA) analyses, time groups (T1 vs T2) exhibited the most important effects over the microbiota composition. The NMDS analysis showed two clear clusters at ASVs, phylum and genus levels (Fig. 1) suggesting a dietary effect on the pig microbiota composition due to the shift from SP to LP between T1 to T2. Group factor (T1 vs T2) explained around 42% of total variability of the relative abundance data. The significance of this finding was confirmed with the PERMANOVA analysis (p < 0.0001), suggesting a strong impact of time-points at all analyzed levels (ASVs, phylum and genus). In addition to the time group effect, other confounding factors such as diet composition or other environmental stressors must coexist to modulate the microbiota composition. For instance, several studies agree that diet is one of the most relevant factors modulating microbiota composition^26,37,38^. In our study, different diets were employed between T1 and T2. Pigs at T1 diet were fed at two protein intakes, whereas at T2 all of them were fed at a low-protein level, although with two different levels of carotenes, a fact that might modulate the composition of microbial gut ecosystem. This dietary modification may also affect environmental conditions and therefore alter microbial populations^11–14^. PERMANOVA analysis was used to assess the effect of the dietary protein at T1 (SP vs LP) and the carotene level at T2 (CE vs NC). Significant effects were observed at genus (p < 0.05) and suggestive at ASVs level (p = 0.07) in T1, whereas no significant effect associated with the carotene level was observed at T2 (p > 0.05). This will confirm that changes in dietary protein play a more relevant effect than carotene level on gut microbiota composition during the last month of fattening. The impact of dietary protein on microbiota composition has been reported in pigs^18,32,33^ and in other mammalian species^15,29,30,39,40^. For example, in mice, lower dietary protein levels reduce the faecal bacterial concentration^30^. Moreover, microbial metabolism of protein-based diet can result in the negative effect of the epithelial cell in the gastrointestinal tract^41^ as product of the formation of deleterious metabolites as phenols, indoles, amines, sulfides, ammonia, and monocarboxylic acids, particularly as a result of fermentation in the large intestine, which are cytotoxic to the cells^36,42^.

### Time and diet-specific modulation of fecal microbiota composition

We also performed a presence-absence (PA) test to identify specific features in T1 and T2 at ASVs, phyla and genus levels (Supplementary Table S1). According to PA, a total of 7 ASVs were only found in T1 and 25 in T2. At phylum and genus levels the PA test did not show any time-point-specific taxa. In addition, a differential abundance (DA) analysis was done to test significant differences in the abundance of features between time-point-groups of animals. The DA between groups (T1 vs T2) identified 6 phyla, 30 genera and 777 ASVs (Supplementary Tables S2, S3 and S4). Amongst the DA phyla, Deferribacteres, Euryarchaeota and Spirochaetes were the most abundant at T1 while Synergistetes, Tenericutes and Spirochaetes were at T2. At genera level, the most abundant at T1 were *Bulleidia*, *Ruminococcus* and *Lachnospira* and at T2, *Treponema*, *CF231* and *RFN20* were identified in a higher proportion.

To identify features linked to the different dietary protein (T1) and pro-Vitamin A (T2) levels, we focused the DA analysis within each time-point. At T1 (SP vs LP), 160 ASVs were detected as DA at false discovery rate of 5%. Within genus, *Dorea* (higher in SP) and *Peptococcus* (higher in LP diet) were DA, whereas no significant differences were found at the phylum level. On the other hand, within T2 group (CE vs NC) a total of 162 ASVs were identified, but only the genus *Epulopiscium* was DA, showing higher abundance in CE. According to DA, a similar number of differentially abundant ASVs was identified at each time-point (160 ASVs, in T1 and 162 ASVs, in T2). However, the overlapping between the ASVs reported as DA across time-points (T1 and T2) was low. For instance, only 12% (20) of ASVs identified as DA in T1 were also reported in T2. Moreover, no overlapping was observed at phylum or genus level. The low overlapping suggests diet-specific gut microbiota modulation as a consequence of the different dietary protein (T1) and carotene (T2) levels.

### Changes in fecal microbiota can discriminate pigs by diet et each time point

**Figure 2:**
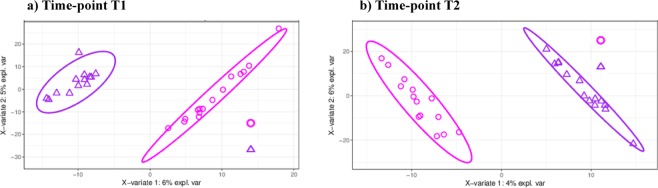
Sparse Partial‐least‐squares Discriminant Analysis plots of T1 group (**a**) and T2 group (**b**) after Amplicon Sequences Variants selection (139 and 151, respectively). The feed factor standard protein (SP) vs low protein (LP) in T1 and carotene-enriched (CE) vs control diet (NC) in T2 factors were used to distinguish groups. At time-point T1, purple color represents the low-protein diet and pink color the standard-protein diet. At time-point T2, purple color represents the carotene-enriched diet and pink color the control diet.

To complement the DA as well as to identify discriminant ASVs which contribute to classify samples according to the protein (SP vs LP) or carotene (CE vs NC) intake, a partial least squares discriminant analysis (PLS-DA) was also implemented. Samples were clearly discriminated by diet using the first component (Fig. 2). The relative abundance of 139 and 151 non-overlapping ASVs were sufficient to classify samples in T1 (Fig. 2a) and T2 (Fig. 2b), respectively.

**Figure 3:**
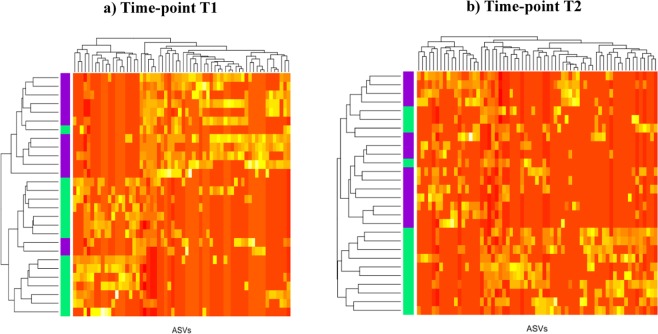
Heat map showing sample distribution using the most commonly identified Amplicon Sequence Variants (ASVs) in the differentially abundant and the sparse partial least squares regression discriminant analyses. At time-point T1, purple color represents the low-protein diet and green color the standard- protein diet. At time-point T2, purple color represents the carotene-enriched diet and green color the control diet.

We then applied a more conservative approach and only retained the ASVs that were simultaneously detected by PLS-DA and DA. In T1, 62 ASVs (44%) (Fig. 3a) were commonly identifies in both analyses. The taxonomic classification of the most abundant features in the SP diet included genera such as *Dorea*, *Oscillospira*, *Faecalibacterium*, *Campylobacter* and *Treponema*. Conversely, the LP diet was associated with a higher abundance of *Anaerovibrio*, *Bulleidia*, *CF231*, *Clostridium*, *Gemmiger*, *Phascolarctobacterium*, *Prevotella*, *Roseburia* and *Succinivibrio*. Noticeably, *Faecalibacterium*, *Treponema*, *Clostridium* and *Prevotella* have been previously reported as sensitive genera to dietary protein in pigs^43^. Thus, in agreement with the observed differences, in pigs, a high-protein diet reduced the abundance of *Faecalibacterium prausnitzii* and *Clostridium coccoides* in cecum and colon^43^, while a low-protein diet increased the proportion of *Prevotella* in cecum^33^.

On the other hand, at T2, 62 ASVs (38%) were commonly identified by both sPLS-DA and DA procedures (Fig. 3b). The taxonomic classification of these ASVs revealed members of *Prevotella*, *Treponema* and *YRC22* amongst the most DA. In humans, *Prevotella* species dominate in the gut microbiome of long-term exposure to low-protein high-fibers diets^44^, Moreover, increased *Prevotella* abundance has been reported in autistic children treated with very high levels of vitamin A^45^. As previously noted, *Prevotella* was among the most discriminant group of bacterial also in T1, were 37% of the AVSs were taxonomic classified as *Prevotella*. In T2, the most represented genus was also *Prevotella* (33%) followed by *Treponema* (8.6%). The ASVs classified as *Prevotella* were most abundant in the LP diet in T1, in line with the pattern described in humans^44^. In contrast, in T2 a non-clear taxonomic pattern between diets (CE or NC) was observed. The lack of evidence for a differential distribution of *Prevotella* genus between CE and NC groups may be explained by the fact that protein intake was the same for these two diets in addition to, as suggested by the PERMANOVA analysis, or by the low impact of dietary carotene on microbiota composition.

### Functional metagenomic prediction of dietary modulation of the fecal microbiota

In order to complement the taxonomic analysis (ASVs, phylum and genus) and to identify functions that may be modulated by the dietary interventions, a metagenomic prediction was done using PICRUSt against the KEGG (Kyoto Encyclopedia of Genes and Genomes) pathways and COGs (Clusters of Orthologous Groups). The metagenomic prediction based on KOs (KEGG Orthology) revealed that globally the gut microbiota was enriched mainly with functions related to membrane transport, metabolism of carbohydrate and amino acid, replication and repair process, translation and energy metabolism (Supplementary Table S5).

For diversity indexes, the Tukey HSD test^46,47^ only led to significant differences for the beta-diversity index (p = 0.002) of predicted KOs between T1 and T2. Similarly to taxonomic abundance, the strongest effect for diversity was associated with time-points (T1 vs T2). We also applied a DA test, identifying a total of 1,277 KOs and 1,470 COGs, which happened to be differentially abundant between T1 and T2. Within each time-point, differences between diets were only observed at T1, with 31 KOs and 47 COGs showing differentially abundance. Again, this result suggests that protein rather carotene intake has a higher impact on microbiota. To gain a deeper insight into their functions, we annotated the KO in it corresponding functional classification module. The 31 KOs belonged to functions mainly related with branched-chain amino acid transport system and NADH: quinone oxidoreductase. Interestingly, the module M00237 (branched-chain amino acid transport system), which was the most overrepresented in SP are directly related with products of protein degradation^48^. This result could be partly explained by the fact that the increased protein intake in SP could trigger the microbial activities related to protein degradation that take place in the colon, where proteolytic bacteria are present^17^.

The global patterns between T1 and T2 for COGs were similar to those found for KOs. The main differences between time groups include functions related with translation, ribosomal structure and biogenesis, cell wall/membrane/envelope biogenesis and transport and metabolism of amino acid (Supplementary Fig. S1). In T1, the main functions associated to the diets were related to the transport and metabolism of amino acids, coenzymes, carbohydrates and lipids (Supplementary Fig. S2). The analysis of the most significant DA COGs at T1 revealed that members of the family fucose permease (COG0738) were more abundant in LP than in SP. Members of this COG family are involved in the fucose uptake, a monosaccharide related to host–microbe interactions^49^. To be noted, recent studies suggest that nitrogen availability is likely to be shaped by host–microbe interactions^29,30^. In addition, long-term reduction of nitrogen have been associated with a higher carbohydrates intake^29^ that may explained the observed higher COG0738 abundance as well as the higher relative abundance of genera associated with carbohydrate metabolism such as *Prevotella*, *Succinivibrio* and *Roseburia* in the group of pigs fed with LP compared to SP diet.

### Medium-term dietary interventions may alter enterotypes stability

**Figure 4:**
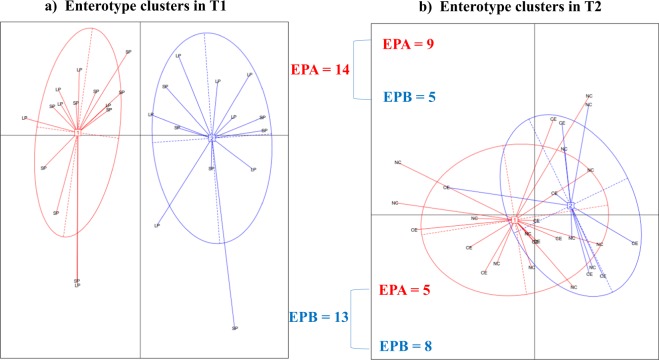
Evolution/transition of pigs from time-point T1 (165 days of age) to time-point T2 (195 days of age). The red color represents EPA: Enterotype like cluster A; the blue color represents EPB: Enterotype like cluster B. CE: carotene-enriched, NC: not carotene, control diet.

In order to evaluate the presence of enterotypes as well as the dietary effect on the enterotype stability, a clustering analysis was done within each time-point. Previous reports have described that there are two main enterotypes in pigs^2,9,10^, unlike humans, where there are at least three dominant enterotypes^50^. In agreement with this, the analysis of our data showed that the optimal number of cluster both at T1 and T2 was two (Fig. 4). The classification in enterotype type A (EPA) and enterotype type B (EPB) was not linked to the diet neither at T1 nor at T2. To characterize the cluster taxonomic composition, a DA was done at genera level. Within T1, EPA and EPB samples showed significantly DA of 19 genera, 17 of which were more abundant in EPA than in EPB. The most DA genera in T1 were *Turicibacter*, *Butyrivibrio* and *Paraeggerthella*, whereas the *Catenibacterium* genus was identified as predominant in EPB (Tables 1 and 2). Interestingly, Lu *et al*. (2018) reported the genus *Turicibacter* as the dominating genus of one of the pig enterotypes in 15-week-old pigs. Similar patterns, in terms of the number of DA genera were observed at T2, with 14 DA genera, 13 of which were more abundant in T2-EPA including *Faecalibacterium*, *Dorea* and *Succinivibrio*. The *Alistipes* genus was the only one that was more abundant in the T2-EPB.

**Table 1:** Genus bacterial differentially abundant between the two predicted enterotype-like clusters in time-point T1 (165 days of age).

Genus	Counts in EPA (SD)	Counts in EPB (SD)	P-values	Adjusted p-value
*Turicibacter*	58.11 (2.47)	8.75 (1.69)	3.09E-03	8.40E-03
*Butyrivibrio*	35.59 (2.70)	0	2.64E-17	1.29E-15
*Paraeggerthella*	28.26 (1.89)	3.19 (0.89)	8.47E-09	4.61E-08
*Asteroleplasma*	28.40 (2.49)	3.78 (1.05)	1.88E-03	5.81E-03
*Victivallis*	20.75 (2.50)	0	1.56E-11	1.53E-10
*Barnesiella*	20.04 (2.46)	0	9.79E-13	1.60E-11
*Megasphaera*	55.34 (3.10)	35.40 (2.90)	2.02E-03	5.81E-03
*L7A_E11*	22.64 (1.97)	3.35 (0.93)	1.65E-02	4.05E-02
*Parabacteroides*	16.82 (2.40)	0	1.81E-12	2.21E-11
*YRC22*	15.72 (2.28)	4.22 (1.17)	7.79E-04	2.94E-03
*Olsenella*	11.49 (1.65)	0	5.58E-11	4.55E-10
*Blautia*	15.77 (2.25)	4.65 (1.29)	1.98E-03	5.81E-03
*rc4-4*	14.39 (1.72)	6.68 (1.27)	3.64E-03	9.38E-03
*Anaeroplasma*	19.56 (2.37)	12.69 (2.38)	1.02E-08	4.98E-08
*Mucispirillum*	7.72 (1.40)	3.10 (0.86)	1.15E-03	4.02E-03
*Selenomonas*	4.32 (1.16)	0	3.78E-09	2.64E-08
*Slackia*	7.72 (1.42)	6.13 (1.18)	4.58E-06	2.04E-05
*Pyramidobacter*	6.87 (1.26)	6.07 (1.68)	1.13E-13	2.76E-12
*Catenibacterium*	16.39 (2.35)	17.50 (2.62)	3.28E-04	1.34E-03

EPA: Enterotype like cluster A; EPB: Enterotype like cluster B; Counts in EPA: log of the Cumulative Sum Scaling (CSS) normalized abundance of each genus and their standard deviation (SD); Counts in EPB: log of the CSS normalized abundance of each genus and their standard deviation (SD); Adjusted p-value: P-value corrected by *q*-value calculated with a false discovery rate approach^68^.

**Table 2:** Genus bacterial differentially-abundant between the two predicted enterotype-like clusters in time-point T2 (195 days of age).

Genus	Counts in EPA (SD)	Counts in EPB (SD)	P-values	Adjusted p-value
*Faecalibacterium*	83.07 (3.29)	7.68 (2.43)	3.74E-05	1.68E-04
*Dorea*	80.27 (2.05)	9.84 (2.09)	1.17E-03	4.17E-03
*Succinivibrio*	122.58 (0.84)	63.08 (0.42)	3.01E-03	7.97E-03
*Butyricicoccus*	61.55 (2.58)	9.83 (2.09)	2.70E-03	7.59E-03
*Anaerovibrio*	100.59 (2.89)	50.72 (2.73)	1.94E-02	4.60E-02
*Megasphaera*	29.28 (2.76)	7.99 (1.79)	1.58E-03	4.81E-03
*Asteroleplasma*	21.75 (2.06)	6.29 (1.50)	1.60E-03	4.81E-03
*Butyrivibrio*	17.40 (1.96)	3.70 (1.17)	1.52E-04	6.22E-04
*Selenomonas*	13.46 (1.77)	0	1.46E-11	3.28E-10
*Catenibacterium*	13.24 (1.76)	0	3.64E-10	4.09E-09
*Barnesiella*	12.49 (2.07)	0	8.72E-13	3.93E-11
*Defluviitalea*	8.59 (1.44)	0	4.62E-09	2.97E-08
*YRC22*	11.60 (1.93)	4.49 (1.42)	1.21E-03	4.17E-03
*Alistipes*	0	10.58 (2.26)	2.67E-07	1.50E-06

EPA: Enterotype like cluster A; EPB: Enterotype like cluster B; Counts in EPA: log of the CSS normalized abundance of each genus and their standard deviation (SD); Counts in EPB: log of the CSS normalized abundance of each genus and their standard deviation (SD); Adjusted p-value: P-value corrected by *q*-value calculated with a false discovery rate approach^69^.

In our study we used two time points (T1 vs T2), therefore, we have the opportunity to test the sample stability in each enterotype across T1 and T2. Within T1, 14 samples clustered in the EPA and 13 in EPB. This classification did not match the protein diet groups. However, at T2 the sample assignation was not as balanced as observed in T1 (Fig. 4). In addition, transitions between the cluster assignations were observed indicating low enterotype stability across time. For instance, only ≈62% of samples remained in the corresponding enterotype in T2 compared to T1 (Fig. 4). In human, the effect of dietary interventions on enterotype resilience has been investigated^51^. In short-term dietary interventions, the microbial community recovers its original status in a few days, including the enterotypes, which remain stable, particularly as compared to long-term perturbations^51^. In healthy pigs, after weaning and under regular management conditions (i.e., diet and absence of antibiotic treatment), the diversity of microbiota increases^6^ which, once the enterotype is established, does not change substantially over time^2,28^. However, in a recent research^10^, working with samples taken at weaning (18 d), 15 weeks after weaning (≈118 d) and 196 d, reported a similar percentage of enterotype shift between samples as we have seen here between 165 d and 195 d. Therefore, the low enterotype stability observed in our study may be explained by the perturbation due to the dietary changes occurred from T1 to T2 as well as the effect of the inclusion of different levels of proteins, the interaction between diet or the age of animals studied. In fact, in line with the enterotype taxonomic composition at T2, the most abundant genera in the SP diet were *Dorea*, *Oscillospira* and *Faecalibacterium*. *Faecalibacterium* is a commensal bacterium, commonly reported in the gastrointestinal tracts of animals and humans as one of the main butyrate producers bacteria in the intestine^52,53^, that has been suggested as a marker of gut health and host wellbeing in humans^54,55^. As mentioned before, in pigs, high-protein diets reduce the proportion of *Faecalibacterium*^43^ and low-protein diets increase the proportion of *Prevotella*^33^. At T2, all pigs were subjected to a low-protein diet, which may explain the observed higher proportion of both *Faecalibacterium* and *Prevotella*. Moreover, co-occurrence (i.e., positive correlations) between both genera^9^ have also been reported in pigs. Supporting previous findings, a moderate positive correlation between *Prevotella* and *Faecalibacterium* was observed, both at T1 (R = 0.44) and T2 (R = 0.26).

To the best of our knowledge, the present study is the first to provide a comprehensive overview of the effect of dietary intervention of protein and pro-Vitamin A carotenoids on the structural and functional composition of pig gut microbiota. Although these factors do not have a relevant impact on community diversity, our results show that dietary protein variations have a stronger effect than carotenes on the compositional and functional structure of the pig gut microbiota. *Prevotella*, *Faecalibacterium*, *Treponema* and *Clostridium* were the genera most influenced by dietary protein, which contributed to the functional classification related with the degradation of protein. Our findings also suggest that enterotype stability can be affected even after medium-term dietary interventions. Additional longitudinal studies are needed to better characterize enterotype stability across fattening, for instance overcoming one of the limitations of our experiment, which was the different duration of protein and carotene interventions. In summary, our results contribute to the knowledge of microbial modulation in pigs and can be useful to study the inclusion of optimal dietary protein to reduce the cost and the environmental impact of pig production without affecting growth performance.

## Method

### Ethics statement

All experimental procedures were approved by the Ethics Committee for Animal Experimentation of the University of Lleida (agreement CEEA 02-04/14) and were performed in accordance with authorization 7704 issued by the Catalan Ministry of Agriculture, Livestock, Fisheries and Food, Spain.

### Animals and experiment design

Feces samples were collected from 32 barrows of a pure Duroc line produced by 23 dams and 7 sires. A detailed description of the management and feeding conditions of these pigs during the experiment have been previously reported^22^. Briefly, all pigs were born within the range of 4 days (d) and castrated within the first week of age. At 70 d of age, they were moved to an experimental research center (Centre d’Estudis Porcins, Torrelameu, Lleida, Spain). There, they were allocated in 8 pens of 4 pigs and raised under identical conditions except the diet (Supplementary Fig. S3). During the growing-finishing phase, from 70 to 165 d of age, a different cereal-based protein diet was given to four alternate pens. Thus, half of the pigs were fed with a standard-protein (SP) diet (17%, 16%, and 15% of crude protein, from 70 d to 110 d, from 110 d to 140 d, and from 140 d to 165 d, respectively) and half with a low-protein (LP) diet (15%, 14%, 13% of crude protein from 70 d to 110 d, from 110 d to 140 d and from 140 d to 165 d, respectively). The 2% differences in protein content are not expected to impact pig performance but can help reduce nitrogen emissions^56^. At the start of the finishing phase, pigs were redistributed across pens so that in each pen there were two pigs fed with the SP diet and two pigs fed with the LP diet. During the finishing phase, from 165 to 195 d, a different cereal-based carotene diet (12% crude protein, without supplemented vitamin A) was given to four alternate pens. The two diets supplied in this phase were identical except for the corn line used in the feed formulation. Thus, half of the pigs were fed with a carotene-enriched (CE) diet (20% of M37W-Ph3 carotenoid-fortified corn)^21^ and half with a control (NC) diet (20% of a near-isogenic M37W corn, which only contains traces of carotenoids). The NC and CE diets were estimated to provide 1,300 IU and at least 2,100 IU of vitamin A/kg. At all phases, sires were equally represented by diet. Fecal samples were collected at 165 d (T1) and 195 d (T2), i.e. at the end of the growing-fattening and finishing phases, respectively, from all the pigs in the experiment. The diet composition in each group (SP vs LP) were formulated following the recommendation of Spanish Foundation for the Development of Animal Nutrition (FEDNA, 2013)^56^. A detailed description of the ingredients and nutrient content of the diets used during the experiment can be found in Supplementary Tables S6 and S7.

### DNA extraction, PCR amplification, and sequencing

Total bacterial DNA from feces was isolated using the DNeasy PowerSoil Kit (QIAGEN, Hilden, Germany), according to the manufacturer instructions with the following modifications: Frozen wet fecal samples (0.1 g) were disrupted with 3 glass beads in a bead homogenizer (BeadMill 4, ThermoFisher) in presence of buffer C1, and incubated at 95 °C for 5 minutes. These steps were repeated twice. Sample tubes were spun at 10,000 g for 30 s and the supernatant transferred to a new tube to follow with the manufacturer instructions. In the final step, the DNA was eluted from the column in a volume of 100 µl. The DNA concentration and quality were estimated by spectrometry (Nanodrop-100) and fluorometry with the dsDNA HS Assay kit (Qubit 4, Invitrogen, Carlsbad, CA). The microbiota composition was assessed by amplifying the V3 region of the 16S rRNA gene using primers adapted from^57^ with NEXTERA dual index specific tails for the forward: 5′TCGTCGGCAGCGTCAGATGTGTATAAGAGACAG and Reverse: 5′GTCTCGTGGGCTCGGAGATGTGTATAAGAGACAG primers. Purified amplicons were paired-end sequenced (2 × 250 nt) on an Illumina MiSeq (Illumina, San Diego, CA, USA) at FISABIO (Valencia, Spain).

### Bioinformatics and statistical analysis of 16S rRNA gene sequences data

The fastq files were processed using QIIME2 software^58^. Cleaned sequencing reads were obtained after remove the primers, barcode sequences, and the low quality reads from the raw data. Paired-end reads were merged, and quality control implementation allowed the retention of sequences with a length of 250 bp and with a mean sequence quality score >30. Sequences with mismatched forward and/or reverse primers were omitted. The cleaned 16S rRNA gene sequences were process into Amplicon Sequences Variants (ASVs) at 99% of identity. All reads were classified to the lowest possible taxonomic rank using QIIME2^58^ and the GreenGenes Database v13.5 as reference. Samples with less than 3000 reads (n = 3) were excluded and not considered in posteriors analysis.

The diversity index of bacterial community was assessed with the Vegan package^59^. Alpha and richness diversity indexes were evaluated with the Chao1^60^ and Shannon^61^ index. Between-sample (beta) diversity was calculated using the unweighted and weighted UniFrac^62^ distances and Bray-Curtis^63^ dissimilarities. Principal-coordinate analysis (PCoA) was used to visualize these distances using Emperor. The zero-inflated Gaussian mixture model implemented in the fitZig function of the MetagenomeSeq R package^64^ was used to identify the ASVs that differed significantly between (T1 and T2) and as function of the diets within each time-points group (SP vs LP, in T1 and CE vs NC, in T2). The Adonis function implemented in Vegan package^59^ was employed to perform a permutational multivariate analysis of variance (9,999 permutations) to the Bray-Curtis^63^ dissimilarities. In addition, to identify a subset of ASVs that discriminate samples according to diets T1 (SP vs LP) and T2 (CE vs NC), Sparse Partial‐least‐squares Discriminant Analysis (sPLS-DA) was implemented using the Mixomics package^65^.

Finally, enterotype-like cluster detection was done using the genera abundance in each sample as described in Arumugam^50^. In brief, samples clusters were detected using the probability distribution distance metric related to Jensen-Shannon divergence and the Partitioning Around Medoids Arumugam^50^. The optimal number of cluster was determined following the Calinski-Harabasz (CH) Index^66^ (R Script available at: http://enterotype.embl.de/enterotypes.html).

### Functional prediction

We explored the functional capacity of the microbiota by inferring metagenomics functionality from the 16S rRNA gene sequencing data using the Phylogenetic Investigation of Communities by Reconstruction of Unobserved States (PICRUSt) software (version 1.1.2)^67^. The corresponding know OTUs (Greengenes v13.5 database) from the ASVs were used to predict the functionality of our sequenced 16S rRNA against the precalculated files corresponding to KEGG (Kyoto Encyclopedia of Genes and Genomes, version 87.1)^68^ pathways and COGs (Clusters of Orthologous Groups).

## Supplementary information

Supplementary Figure S1,S2 and S3

Supplementary Table S1. Amplicon Sequences Variants (ASVs) identified in the presence-absence test between time-points for ASVs

Supplementary Table S2. Amplicon Sequences Variants (ASVs) identified in the differentially abundant test between time-points for ASVs.

Supplementary Table S3. Amplicon Sequences Variants (ASVs) identified in the differentially abundant test between time-points for phyla

Supplementary Table S4. Amplicon Sequences Variants (ASVs) identified in the differentially abundant test between time-points for genera

Supplementary Table S5. Functional modules of Kyoto Encyclopedia of Genes and Genomes Orthology (KOs) identified as differentially abundant

Supplementary Tables S6 and S7. Detailed description of the ingredients and nutrient content of the diets used during the experiment.

Supplementary Dataset 8. Legend of Amplicon Sequences Variants

## Data Availability

All data supporting the findings of this study are available within the article and Supplementary Information, or are available from the corresponding author upon reasonable request.
