# Supplementary material for: Modulatory Effect of Protein and Carotene Dietary Levels on Pig gut Microbiota
Source: Sci Rep. 2019 Oct 10;9:14582. doi: 10.1038/s41598-019-51136-6 (PMC6787051; doi:10.1038/s41598-019-51136-6)
Supplement: Supplementary file 1 — Supplementary Figure S1,S2 and S3 [file 41598_2019_51136_MOESM1_ESM.pdf]

Supplementary Figures

Modulatory Effect of Protein and Carotene Dietary Levels on Pig gut Microbiota

Rayner González-Prendes[1,2], Ramona Natacha Pena[1], Emma Solé[1], Ahmad Reza Seradj[1], Joan Estany*[1], Yuliaxis Ramayo-Caldas[3]

[1] Departament de Ciència Animal, Universitat de Lleida-Agrotecnio Centre, Lleida 25198, Catalonia, Spain. [2]Animal Breeding and Genomics Group, Wageningen University & Research, Droevendaalsesteeg 1, 6708 PB. Wageningen, The Netherlands. [3]Animal Breeding and Genetics Program, IRTA, Torre Marimon, Caldes de Montbui, Catalonia, Spain. *Correspondence: jestany@ca.udl.cat

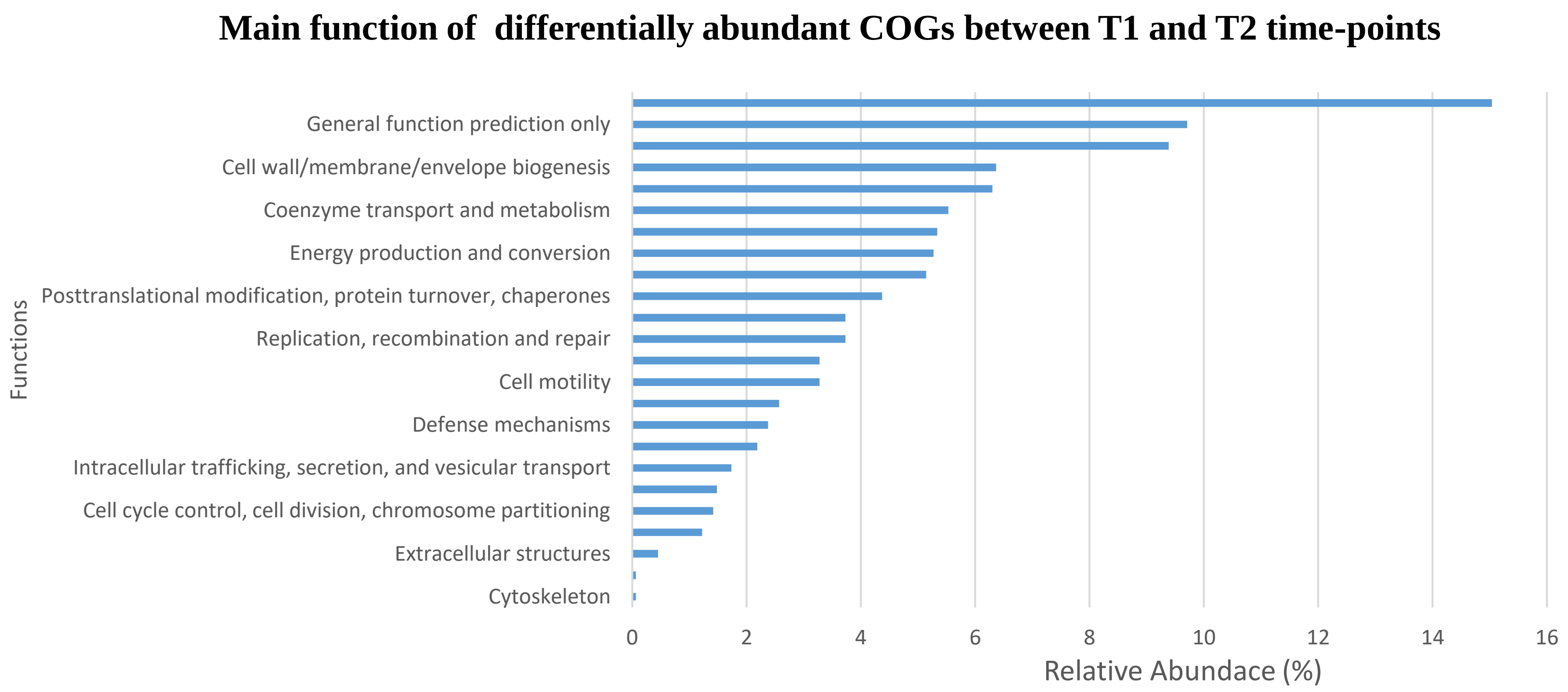

Supplementary Figure S1. Metagenome functional prediction of Clusters of Orthologous Groups (COGs) identified as differentially abundant between time-points T1 (165 days of age) and T2 (195 days of age).

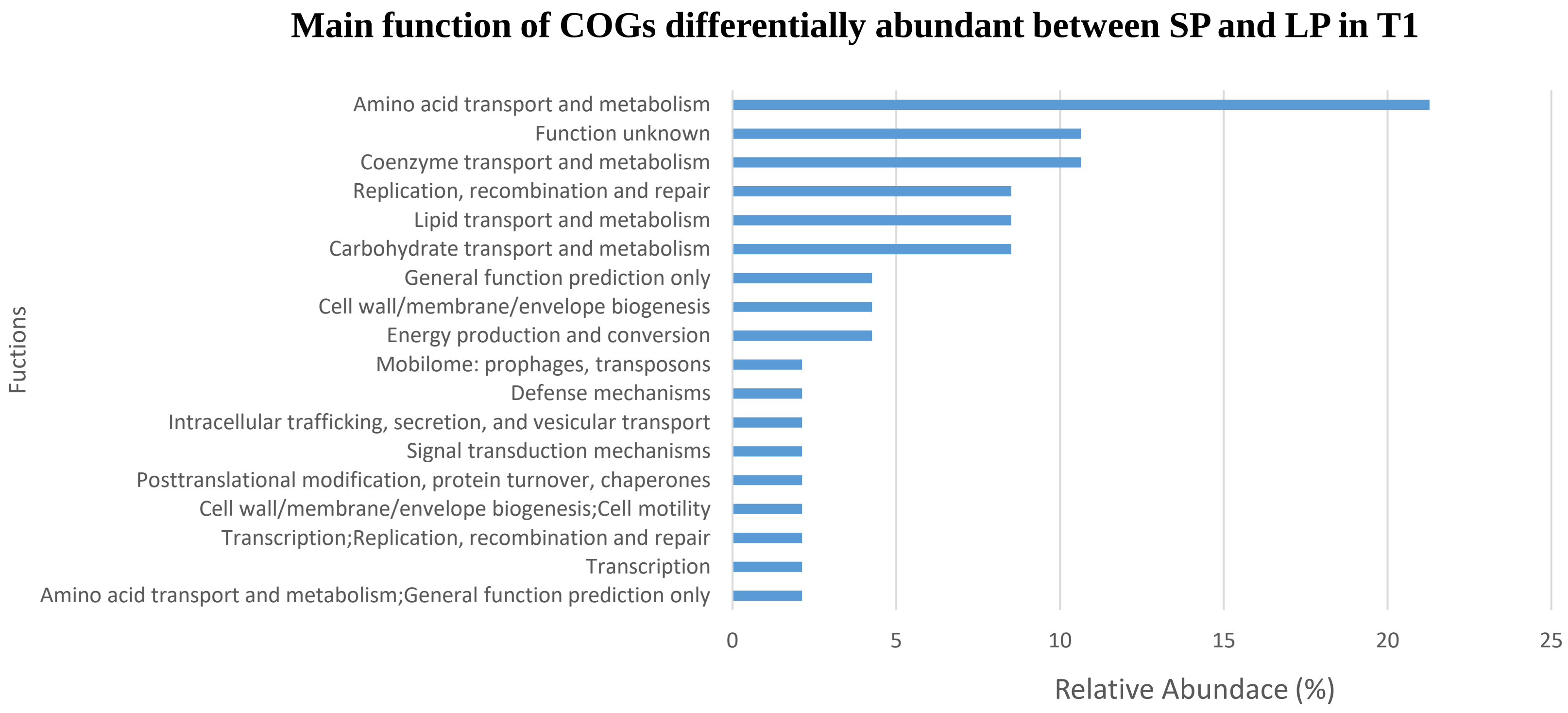

Supplementary Figure S2. Metagenome functional prediction of Clusters of Orthologous Groups (COGs) identified as differentially abundant between standard-protein (SP) and low-protein (LP) diet at time-point T1 (165 days of age).

Experimental design

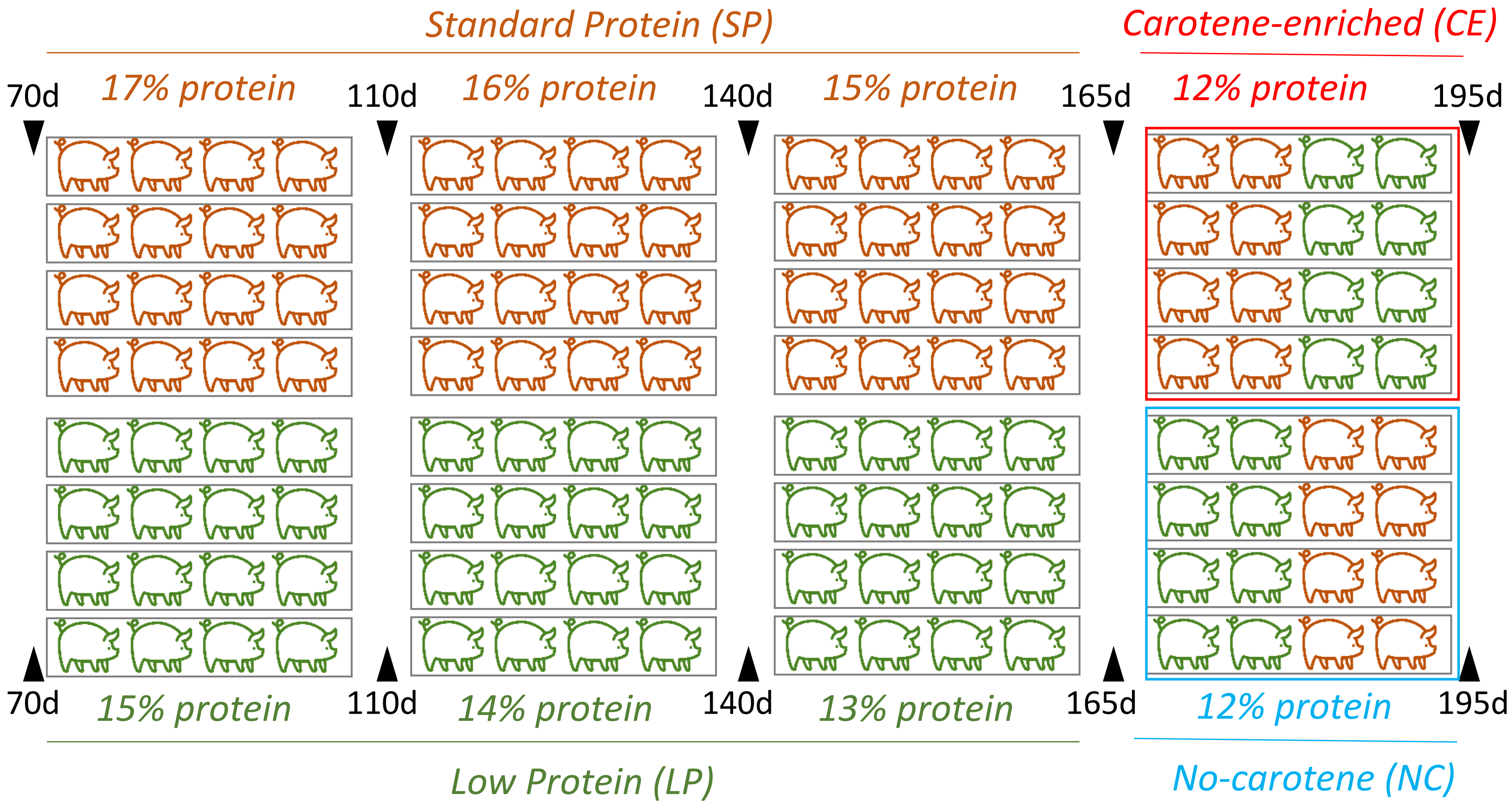

Supplementary Figure S3. Schematic representation of the experimental design. Fecal samples were collected from all pigs at two time points: T1 (165 days of age) and T2 (195 days of age).
