# Supplementary material for: Modulatory Effect of Protein and Carotene Dietary Levels on Pig gut Microbiota
Source: Sci Rep. 2019 Oct 10;9:14582. doi: 10.1038/s41598-019-51136-6 (PMC6787051; doi:10.1038/s41598-019-51136-6)
Supplement: Supplementary file 7 — Supplementary Tables S6 and S7. Detailed description of the ingredients and nutrient content of the diets used during the experiment. [file 41598_2019_51136_MOESM7_ESM.pdf]

Supplementary Tables S6 and S7

Modulatory Effect of Protein and Carotene Dietary Levels on Pig gut Microbiota

Rayner González-Prendes[1,2], Ramona Natacha Pena[1], Emma Solé[1], Ahmad Reza Seradj[1], Joan Estany*[1], Yuliaxis Ramayo-Caldas[3]

[1] Departament de Ciència Animal, Universitat de Lleida-Agrotecnio Centre, Lleida 25198, Catalonia, Spain. Animal Breeding and Genomics Group, Wageningen University & Research, Droevendaalsesteeg 1, 6708 PB. Wageningen, The Netherlands. [3]Animal Breeding and Genetics Program, IRTA, Torre Marimon, Caldes de Montbui, Catalonia, Spain. *Correspondence: jestany@ca.udl.cat

Supplementary Table S6. Diet in the growing-finishing phase of experiment from 70 to 165 days of age corresponding to T1 time group[1].

Item	Feeding stage					
	I (70 to 110 days of age)		II (110 to 140 days of age)		III (140 to 165 days of age)	
	LP	SP	LP	SP	LP	SP
Barley	18.160	14.438	38.000	12.000	21.669	15.000
Wheat	40.000	40.000	-	30.000	20.000	20.000
Triticale	5.484	3.208	-	10.000	-	12.106
Maize	10.000	10.000	27.385	9.840	25.000	11.712
Bakery byproducts	6.000	6.000	12.000	10.000	11.000	11.000
Rapeseed meal 00	-	-	-	10.000	3.670	10.000
Soybean meal 47	7.102	13.312	11.833	7.962	1.128	2.356
Sunflower meal	-	-	-	-	6.000	5.674
Enersoy 3600	5.000	5.000	-	-	-	-
Arroz cilindro	-	-	3.642	5.000	6.000	6.000
Sugar beet pulp	3.000	3.000	0.800	-	-	-
Soybean oil	1.091	1.403	-	-	-	-
Blended animal-vegetable fat 3/5	-	-	1.458	1.896	1.513	2.852
Calcium carbonate	1.209	1.196	1.300	1.008	1.070	0.986
Monocalcium phosphate	0.682	0.628	0.646	0.545	0.339	0.229
Sepiolite	-	-	0.612	-	0.500	0.500
Vitamin-mineral premix[1]	0.400	0.400	0.500	0.500	0.500	0.500
Sodium Bicarbonate	-	-	0.321	0.216	0.261	0.048
Sodium chloride	0.377	0.376	0.414	0.208	0.200	0.294
L-lysine, CP 50%	0.997	0.707	0.673	0.586	0.836	0.614
DL-Methionine, 88%	0.225	0.155	0.170	0.069	0.067	0.011
L-Valine	-	-	-	-	0.031	-
L-Threonine	0.243	0.159	0.190	0.139	0.165	0.094
L-Tryptophan	0.026	0.015	0.039	0.014	0.034	0.009

[1] I- The vitamin and mineral premix for pigs between 70 to 110 days of age contained (per kg of complete diet): 8000 IU of vitamin A; 800 IU of vitamin D3;40 mg of α-tocopherol; 2.4×10^{-2}mg of vitamin B12; 0.8 mg of vitamin B1; 1.6 mg of vitamin B6; 4 mg of vitamin B2; 1.2 mg of vitamin K3; 16 mg of nicotinicacid; 8 mg of pantothenic acid; 280 mg of choline chloride; 0.08 mg of biotin; 0.4 mg of folic acid; 72 mg of Fe (FeCO3); 0.32 mg of I (KI); 0.16 mg of Co(CoSO4•7H2O); 128 mg of Cu (CuSO4•5H2O); 23.8 mg of Mn (MnO); 80 mg of Zn (ZnO); 0.24 mg of Se (Na2O3Se); 0.264 mg of citric acid; 600 FYT 6-phytase;2000 BGU of endo-(1,4)-β-glucanase; 4800 FXU of endo-(1,4)-β-xylanase; 0.264 mg of ethoxyquin.

III- The vitamin and mineral premix for pigs between 110 to 140 days of age contained (per kg of complete diet): 6,250 IU of vitamin A; 1,920 IU of vitaminD3; 14.4 mg of α-tocopherol; 1.7×10^{-2}mg of vitamin B12; 1.44 mg of vitamin B6; 3.84 mg of vitamin B2; 17.28 mg of nicotinic acid; 8.64 mg of calciumpantothenate; 36 mg of choline chloride; 16.6 mg of betaine anhydrous; 96 mg of Fe (FeCO3); 0.96 mg of I (KI); 0.19 mg of Co (2CoCO33Co(OH)2•H2O);14.4 mg of Cu (CuSO4•5H2O); 48 mg of Mn (MnO); 105.6 mg of Zn (ZnO); 0.97% CaCO3; 0.21 mg of Se (Na2O3Se); 1.92 mg of butyl-hydroxytoluene; 6.62 mgof citric acid; 0.19 mg of sodium citrate; 192 mg of sepiolite; 480 FTU of 6-phytase; 0.5 g of Belfeed B 220® (Beldem, Groot Bijgaarden, Belgium) (xylanase) and 2 g of Toxidex® (Virbac, Espluges de Llobregat, Barcelona, Spain) (mycotoxin inhibitor); 10 g of Rehydra Pro® (Adiveter S.L. Reus, Tarragona, Spain)(organic acids and surfactant).

III- The vitamin and mineral premix for pigs between 140 to 165 days of age contained (per kg of complete diet): 6500 IU of vitamin A; 2000 IU of vitaminD3; 15 mg of α-tocopherol; 1.8×10^{-2}mg of vitamin B12; 1.5 mg of vitamin B6; 4 mg of vitamin B2; 18 mg of nicotinic acid; 9 mg of calcium pantothenate;37.5 mg of choline chloride; 17.28 mg of betaine anhydrous; 100 mg of Fe ($FeCO_3$); 1 mg of I (KI); 0.198 mg of Co ($2CoCO_33Co(OH)_2 \cdot H_2O$); 15 mg of Cu($CuSO_4 \cdot 5H_2O$); 50 mg of Mn (MnO); 110 mg of Zn (ZnO); 0.97% $CaCO_3$; 0.22 mg of Se (Na_2O_3Se); 2 mg of butyl-hydroxytoluene; 6.9 mg of citric acid; 0.2 mgof sodium citrate; 200 mg of sepiolite; 500 FTU of 6-phytase; 0.5 g of Belfeed B 220® (Beldem, Groot Bijgaarden, Belgium) (xylanase) and 2 g of Toxidex®(Virbac, Espluges de Llobregat, Barcelona, Spain) (mycotoxin inhibitor).

Supplementary Table S7. Diet in the finishing phase from 165 to 195 days of age corresponding to T2 time group[1].

**Item**<br>Ingredients	%
Barley	57.15
Rapeseed meal	9.03
Wheat shorts	5.26
Corn[1]	20.66
Blended animal: vegetal fat (3:5)	3.96
Monocalcium phosphate	0.94
Salt	0.89
Calcium carbonate	0.72
L-Lysine (50%)	0.54
Dl-Methionine (99%)	0.07
L-Threonine	0.07

[1]The enriched-carotene diet used the fortified corn line M37W-Ph3 (7.0 ug/g DM of β-carotene) whereas the control diet used its near isogenic corn line M37W (nondetected β-carotene content).
